# Nonlinear Statistical Analysis of Normal and Pathological Infant Cry Signals in Cepstrum Domain by Multifractal Wavelet Leaders

**DOI:** 10.3390/e24081166

**Published:** 2022-08-22

**Authors:** Salim Lahmiri, Chakib Tadj, Christian Gargour

**Affiliations:** 1 Department of Supply Chain and Business Technology Management, John Molson School of Business, Concordia University, Montreal, QC H3G 1M8, Canada; 2 Department of Electrical Engineering, École de Technologie Supérieure, Montreal, QC H3C 1K3, Canada

**Keywords:** infant cry signal, expiration, inspiration, cepstrum, multifractal wavelet leaders, multifractal spectrum, cumulants

## Abstract

Multifractal behavior in the cepstrum representation of healthy and unhealthy infant cry signals is examined by means of wavelet leaders and compared using the Student *t*-test. The empirical results show that both expiration and inspiration signals exhibit clear evidence of multifractal properties under healthy and unhealthy conditions. In addition, expiration and inspiration signals exhibit more complexity under healthy conditions than under unhealthy conditions. Furthermore, distributions of multifractal characteristics are different across healthy and unhealthy conditions. Hence, this study improves the understanding of infant crying by providing a complete description of its intrinsic dynamics to better evaluate its health status.

## 1. Introduction

The complexity of various physiological signals is mainly due to the associated complex nonlinear dynamical processes [1]. Therefore, the complete characterization of nonlinear variations in a given physiological signal would help to understand the differences between healthy and pathological cases. For instance, this could be achieved by quantifying the changes in a physiological signal complexity due to abnormalities in terms of variations in its measures.

One of the most useful nonlinear analysis techniques for complex and dynamical systems is multifractal analysis. Indeed, fractal structures are characterized by self-similarity measured by a scaling-independent exponent obtained from a power or scaling law [2]. Therefore, power laws are prevailing analytical techniques used to measure self-similar and the scaling properties of information contents in biological signals. Specifically, in recent years, multifractal analysis has received growing attention in biomedical engineering. For example, it was applied in the analysis of various biophysiological signals, including EEG [3,4,5,6,7,8,9,10], ECG [11,12,13,14,15], magnetic resonance images and brainstem volume [16,17,18], mammograms [19,20], bone radiographic images [21], retina digital images [22], dental implant ultrasonic signal [23], and liver tissue images [24], to name few.

The main purpose of the current work is to measure multifractal properties in healthy and pathological infant cry signals to obtain multiscale distinctive biomarkers. The advantage of multifractal formalism is to obtain more exponents. In particular, the signal under study is divided into several fractal sets, each producing a fractal dimension. The generated fractals are represented in a spectrum of exponents, where each scale is associated with a specific fractal dimension. Indeed, such a representation contains a full description of nonlinear dynamics in the original, for instance, information regarding short and long variations in the signal.

In addition to measuring multifractal properties in healthy and pathological infant cry signals, we sought to investigate whether such measures of multifractal properties are statistically different between healthy and pathological infant cry signals. In this regard, we measured multifractal properties using the multifractal wavelet leaders (MFWL) technique [25] thanks to its intrinsic merits in terms of computational cost and efficiency compared to multifractal detrended fluctuation analysis (MFDFA) [26] and the generalized Hurst exponent (GHE) [27].

Since the acoustical analysis of infant cry signals is independent of human involvement and useful in evaluating pain in pediatric wards, recent works have used various features to characterize infant cry signals, including, melody and short-term rhythm features; mel-frequency cepstral coefficients [28,29,30]; standard acoustic parameters [31]; fundamental frequency glide and resonance frequency dysregulation [32]; resonance frequency averages, durations, and transitions [33]; and wavelet packet transform-based energy and entropies [30]. Furthermore, we believe that measuring multifractal properties of a baby cry record is a valid approach to studying its nonlinear structure so as to better understand its physiology. Hence, in the present paper, we report multifractal analysis from the cepstrum [34,35] of infant cry, where each fractal quantifies the variability in the scaling of the fluctuations in the underlying cepstrum. Indeed, the cepstrum is widely employed in audio signal analysis, as it provides a description of the spectrum envelope and spectral richness and characterizes the harmonic and noise components of the original signal [36]. In this regard, we make the hypothesis that the subtle variations in the cepstrum can be related to the variations in the original cry dynamics. This hypothesis will be verified by performing a formal statistical test, for instance, the Student-*t* test for equality of means between multifractal spectra of healthy and pathological infants’ cepstrum domain.

It should be kept in mind that common works on infant crying records have focused on the extraction of standard acoustic patterns and the usage of standard machine learning models for classification purposes [28,29,30,31,32,33]. Furthermore, most recent studies employed deep learning models to achieve very accurate classification between healthy and unhealthy baby cry records [37,38].

Our contributions to the literature on infant cry signal analysis [28,29,30,31,32,33,37,38] are summarized as follows. To the best of our knowledge, our paper is the first to report a study in which measures derived from the multifractal spectrum of infant cry signals can be used as a promising tool in the analysis of abnormalities in infant cry signals. Such analysis is followed by a rigorous statistical inference to check whether or not multifractal spectra are statically different across healthy and unhealthy infant cries. In this regard, our findings are expected to help improve our understanding of the nonlinear dynamics in healthy and pathological cries for better diagnosis.

The rest of the paper is organized as follows. Section 2 presents the methods. Section 3 describes the data and provides the results. Finally, the conclusion is provided in Section 4.

## 2. Materials and Methods

**Figure 1:**
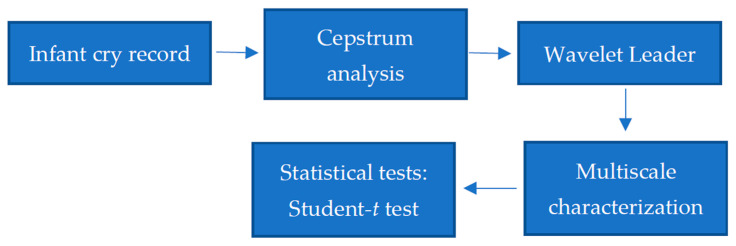
Flowchart of nonlinear analysis of infant cry records.

To evaluate the discriminative power of multifractal proprieties in distinguishing between healthy and pathological infant cry signals, we follow a methodology based on three steps. First, Fourier analysis is applied to each infant cry signal to obtain its corresponding cepstrum used to describe the spectral richness and harmonic characteristics of the original infant cry signal. Second, multifractal analysis is applied to each cepstrum to extract its multiscale Hurst exponent used to describe multiscale nonlinear cepstrum dynamics. In this regard, the multifractal wavelet leaders (MFWL) method is employed to obtain a multiscale spectrum that characterizes the cepstrum of healthy and unhealthy cry records. Finally, in the third step, the Student *t*-test will be performed to check whether or not multifractal descriptors are different across healthy and pathological cepstra. The cepstrum and multifractal analyses are described next. Figure 1 shows the flowchart of our approach for nonlinear analysis of healthy and unhealthy infant cry records.

### 2.1. Cepstrum Analysis

Cepstral analysis was initially developed in the field of homomorphic deconvolution [34,35]. For instance, the complex cepstrum $\hat{s}\left(n\right)$ of a signal *s*(*n*) is given by the inverse Fourier transform of its log spectrum as follows:

(1) $$\hat{s}\left(n\right)=\frac{1}{2\pi }\overset{\pi }{\underset{-\pi }{\int }}\ln S\left(e^{J\omega }\right)\;e^{J\omega n}d\omega$$

where $S\left(e^{J\omega }\right)$ is the discrete Fourier Transform of *s*(*n*) and is given by:

(2) $$S\left(e^{J\omega }\right)=\sum_{n=-\infty }^{\infty }s\left(n\right)e^{-J\omega n}=\left|S\left(e^{J\omega }\right)\right|e^{J\theta \left(\omega \right)}$$

where $\left|S\left(e^{J\omega }\right)\right|$ and *θ*(*ω*) are, respectively, the amplitude and the phase spectra.

Furthermore, the real cepstrum of a signal takes into consideration only its amplitude spectrum. Specifically, the real cepstrum $\hat{r}\left(n\right)$ of a signal *s*(*n*) is given by:

(3) $$\hat{r}\left(n\right)=\frac{1}{2\pi }\overset{\pi }{\underset{-\pi }{\int }}\ln\left|S\left(e^{J\omega }\right)\right|\;e^{J\omega n}d\omega$$

In this study, the real cepstrum is calculated from healthy and pathological infant cry records. Then, multifractal properties are estimated from the obtained real cepstra to characterize healthy and pathological infant cry records. The multifractal descriptors are estimated using the multifractal wavelet leaders (MFWL) [25].

### 2.2. Wavelet Leaders

The multifractal analysis method based on wavelet leaders [25] relies on the discrete wavelet transform to describe the characteristics of the singularity spectrum on a full domain while having solid theoretical and mathematical foundations. For instance, the discrete wavelet transform of signal *X* = {*x_k_*, *k*∈*Z*} is given by:

(4) $$d_{x}\left(j,k\right)=\underset{R}{\int }X\left(t\right)2^{-j}\psi_{0}\left(2^{-j}t-k\right)dt$$

where *ψ*_0_ is a mother function with compact time support, *t* is time script, *j* is parameter of dilation scale, and *k* is parameter of translation. Then, for signal *X*, let *S*(*q*,*j*) denote the structure function and ζ(*q*) the scaling exponents, where *q* is the order (or moment) of multi-resolution. They are expressed as follows:

(5) $$S\left(q,j\right)=\frac{1}{n_{j}}\overset{n_{j}}{\underset{k=1}{\sum }}{\left|L_{X}\left(j,k\right)\right|}^{q}$$

(6) $$\zeta \left(q\right)=\underset{j\to 0}{lim}\;inf\left(\frac{\log S\left(q,j\right)}{j}\right)$$

where *L_X_* represents the largest wavelet coefficient calculated at all finer scales. The multifractal spectrum *D*(*h*) is obtained by the Legendre transform of the scaling exponents. It is defined in the following way:

(7) $$D\left(h\right)=\underset{q\neq 0}{inf}\left(1+qh-\zeta \left(q\right)\right)$$

The information about the variability of the regularity of the signal *X* is described by singularity (or multifractal) spectrum *D*(*h*) defined as the Hausdorff dimension as a function of Hölder (Hurst) exponent that takes the value *h*. Recall that the larger the width of *D*(*h*) is, the more pronounced the level of multifractality/complexity in the signal. The scaling exponents can be computed as:

(8) $$\zeta \left(q\right)=\overset{\infty }{\underset{p=1}{\sum }}c_{p}\frac{q^{p}}{p!}$$

where the log cumulants *c_p_* satisfy ∀*p* ≥ 1, *C*(*j*,*p*) = *c*_0,*p*_ + *c_p_* log(2*^j^*), and *C*(*j*,*p*) is the cumulant of order *p* ≥ 1 of the random variable log(*L_X_*(*j*,⋅)).

In practice, ζ(*q*) and *c_p_* can be estimated by linear regressions as:

(9) $$\left(q\right)=\underset{j}{\sum }w_{j}\log\left(S\left(j,q\right)\right)$$

(10) $$c_{p}=\log\left(e\right)\underset{j}{\sum }b_{j}C_{p}\left(j\right)$$

for scales *j* with classical linear regressions weights *w* (Equation (9)) and *b* (Equation (10)). The cumulants are suitable measurements of the scaling exponents ζ(*q*) [25] and are good descriptors of multifractal properties of the signal understudy. Specifically, cumulants are able to highlight the difference between mono- and multifractal processes [25]. Specifically, the first cumulant captures linearity in the spectrum, whilst the second and the third cumulant capture deviations from linearity.

In the current study, the biorthogonal wavelet is employed as the mother function, the number of scales *j* is set to three, and the *q*-moment varies from −5 to +5. The biorthogonal wavelet is chosen thanks to its symmetry and regularity, which are useful for signal analysis. The scale is set to three as it is suitable to detect slowly varying dynamics for better characterization of the signal. Finally, there is no formal rule on how to set the number of *q*-moments. In this study, it is set to vary from −5 to +5, which is suitable for the number of data points in the obtained cepstra, i.e., 1000.

## 3. Results

The database is composed of two sets: the expiration (EXP) set and the inspiration (INS) set. The EXP set has 2638 cry signals and the INS set has 1860 cry signals. Specifically, there are 1319 healthy signals and 1319 unhealthy signals in the EXP set. Additionally, there are 930 healthy signals and 930 unhealthy signals in the INS set. To record cry signals, a two-channel sound recorder with a sampling frequency of 44.1 kHz and a resolution of 16 bits was placed at 10 cm to 30 cm from the infant. The time duration of each recorded signal is 2–3 min. Each original recorded cry signal was pre-processed to remove background noise and artifacts. It was also segmented to keep only respiration and expiration episodes. The segmentation task was manually performed using the Wave Surfer tool.

All infant cry signals were recorded in the neonatology departments of the following hospitals: Sainte-Justine hospital (Montreal, QC, Canada), and Al-Sahel and Al-Raee hospitals, both in Lebanon. The infants who entered the study were preterm and full-term, and their respective ages ranged from 1 to 53 days. The sample includes both healthy and unhealthy babies and both males and females. The group of unhealthy babies were suffering from various pathologies, such as diseases affecting the central nervous system and the respiratory system. Other pathologies include blood disorder, chromosomal abnormality, and congenital cardiac anomaly. It is worth mentioning that all statistical tests (Student *t*-test) are performed at a 5% significance level.

**Figure 2:**
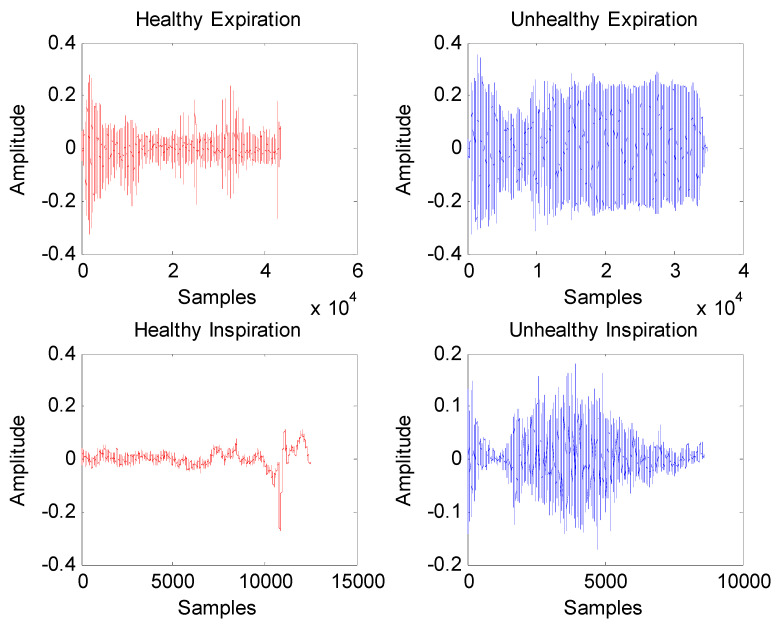
Example of healthy and unhealthy cry signals.

**Figure 3:**
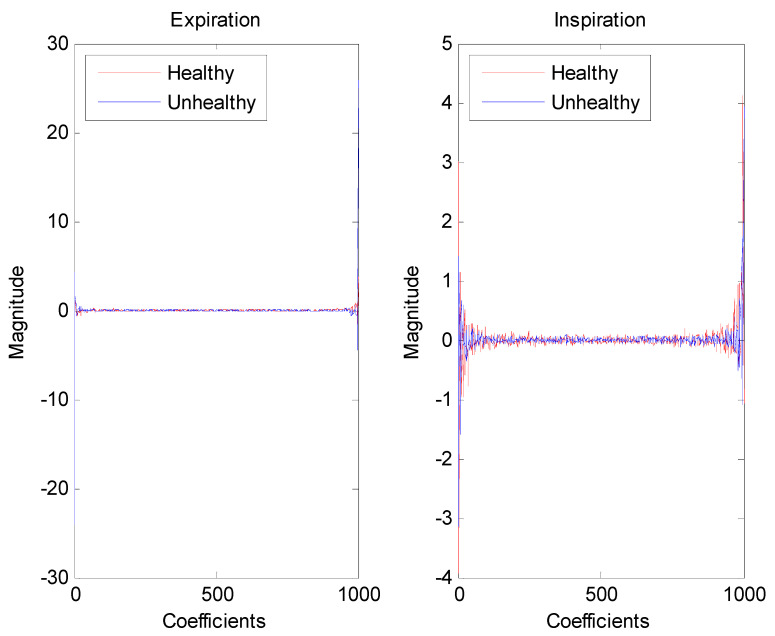
Examples of cepstra from healthy and unhealthy infant cry signals.

For illustration purposes, Figure 2 displays examples of healthy and unhealthy signals. As seen, the length of the infant cry signal is very large; hence, applying Fourier analysis to obtain its corresponding cepstrum will not only help to extract its intrinsic oscillations but also considerably help to reduce its dimensions for further processing by multifractal analysis. For instance, plots of cepstra representing healthy and unhealthy cry signals are shown in Figure 3, where each cepstrum has a size of 1000 data points. As shown, in Figure 3, the cepstrum of an unhealthy infant cry record displays more variability than that of healthy infant cry records.

**Figure 4:**
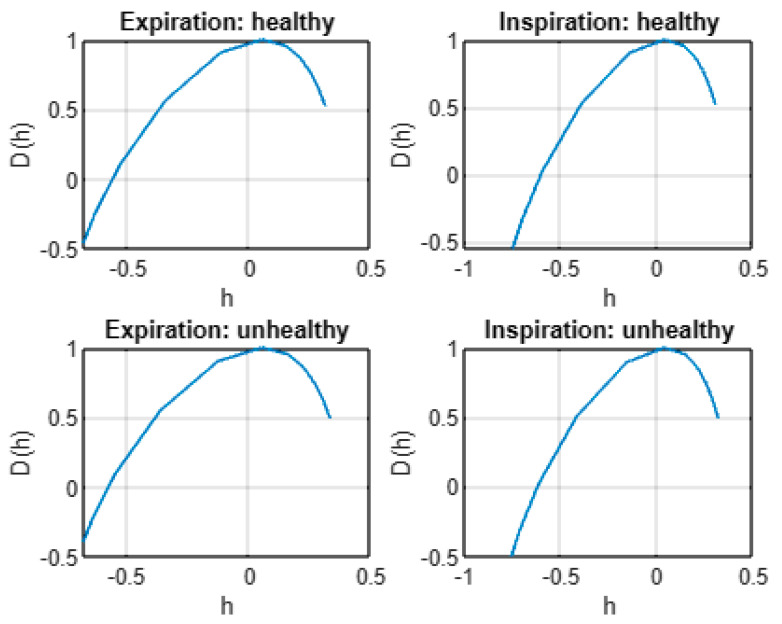
Average multifractal spectra *D*(*h*) of cepstra from healthy and unhealthy infant cry signals.

**Figure 5:**
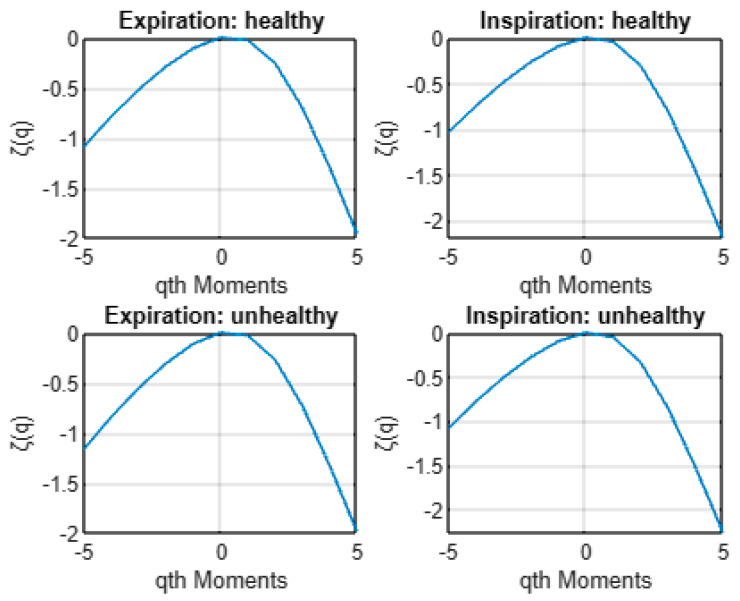
Average scaling exponent function ζ(*q*) of cepstra from healthy and unhealthy infant cry signals.

Figure 4 shows the plot of the average multifractal spectrum *D*(*h*) for healthy and unhealthy infants following expiration and inspiration. As shown, in all situations, the average *D*(*h*) describes a nonlinear form as a function of *h*. Hence, the cepstra of healthy and unhealthy infant cry signals exhibit multifractal characteristics under both expiration and inspiration phases. Furthermore, Figure 5 exhibits the average estimated scaling exponent function ζ(*q*) of cepstra of healthy and unhealthy infant cry signals. As shown, the average scaling exponents ζ(*q*) are a nonlinear function of the moments *q* for both expirations and inspirations under both healthy and unhealthy conditions. These findings confirm the multifractal behavior in cepstra as revealed first by the average multifractal spectrum *D*(*h*).

**Figure 6:**
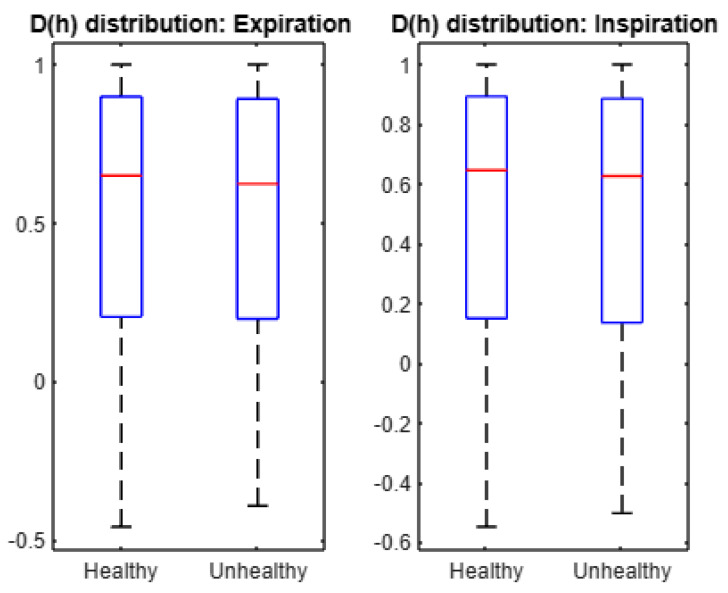
Boxplots of the average multifractal spectra *D*(*h*).

For statistical analyses, we display the box plot of the average multifractal spectra in Figure 6. We performed one-tailed Student *t*-test to verify the null hypothesis that the mean of *D*(*h*) in a healthy set is larger than that in an unhealthy set. Accordingly, we found that the mean of *D*(*h*) in healthy expirations is larger than the mean of *D*(*h*) in unhealthy expirations (*p*-value = 0.4990). Similarly, we found that the mean of *D*(*h*) in healthy inspirations is larger than the mean of *D*(*h*) in unhealthy inspirations (*p*-value = 0.4990). This finding suggests that the mean of spectrum *D*(*h*) is larger under healthy conditions than under unhealthy conditions. In addition, the width of the average multifractal spectrum (*D*(*h*)) is 0.9816 for healthy expirations and 0.8820 for unhealthy expirations. Thus, the cepstra of healthy expirations show a larger degree of multifractality than unhealthy expirations. In addition, the width of the average multifractal spectrum (*D*(*h*)) is 1.0694 for healthy inspirations and 0.9956 for unhealthy inspirations. Hence, the cepstra of healthy inspirations show a larger degree of multifractality than unhealthy inspirations. In summary, expiration and inspiration signals exhibit more complexity under healthy conditions than under unhealthy conditions.

**Figure 7:**
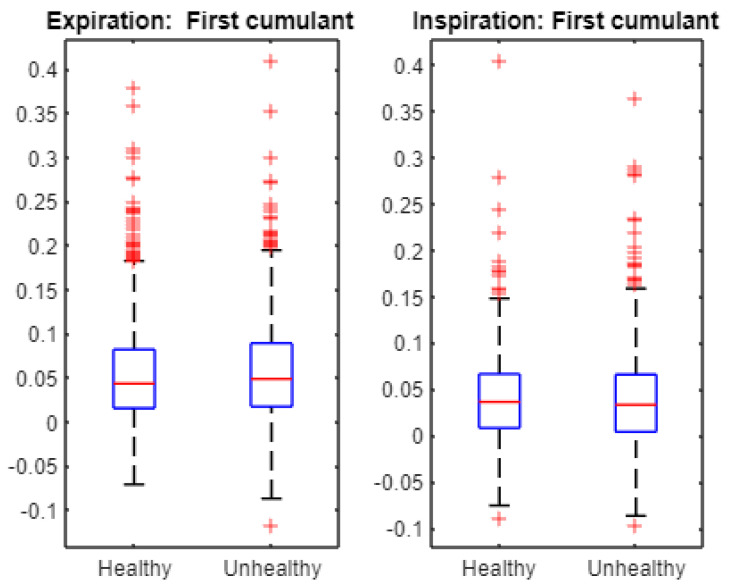
Multifractal wavelet leaders: first cumulant boxplots. For both expiration and inspiration sets, the Student *t*-test indicates that the first cumulant is statistically different across healthy and unhealthy infant cry signals at the 5% statistical level.

**Figure 8:**
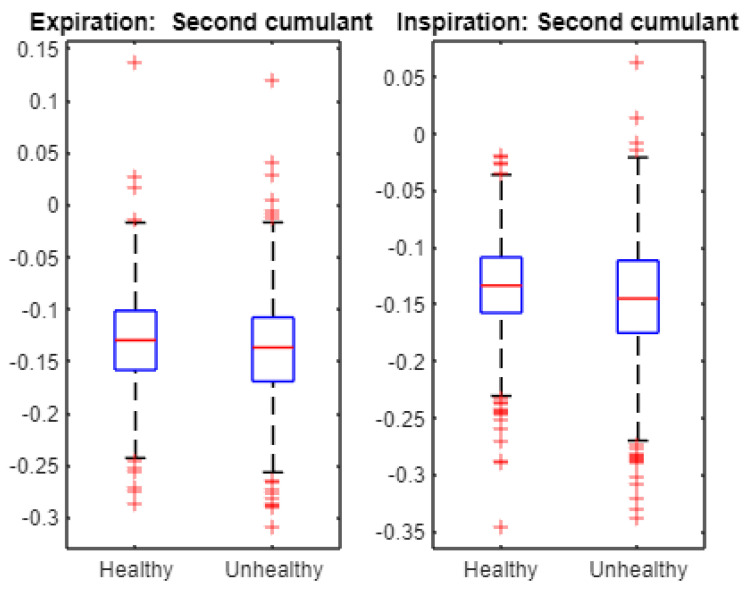
Multifractal wavelet leaders: second cumulant boxplot. For both expiration and inspiration sets, the Student *t*-test indicates that the second cumulant is statistically different across healthy and unhealthy infant cry signals at 5% statistical level.

**Figure 9:**
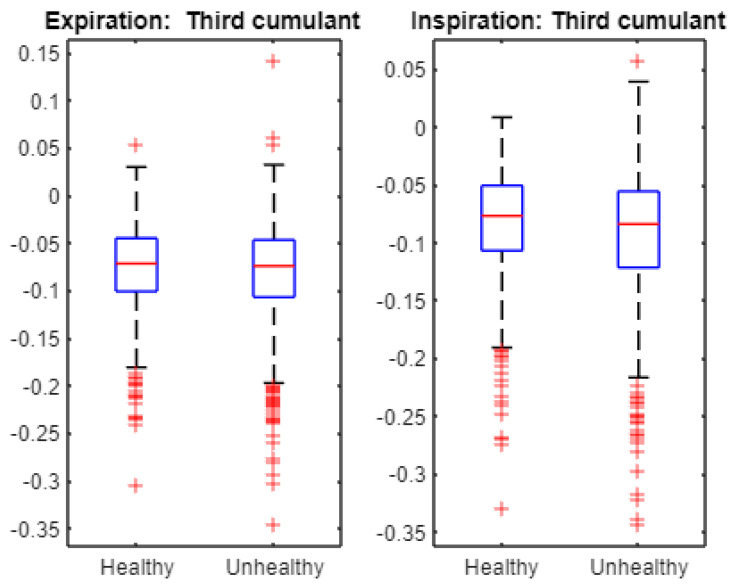
Multifractal wavelet leaders: third cumulant boxplot. For both expiration and inspiration sets, the Student *t*-test indicates that the third cumulant is statistically different across healthy and unhealthy infant cry signals at the 5% statistical level.

Finally, we statistically examined the cumulants to characterize the cepstra of infant cries. For instance, Figure 7 displays the first cumulant boxplots. The Student *t*-test accepts the null hypothesis of equality of the means when applied to first cumulant data from healthy and unhealthy expirations as the associated *p*-value is 0.2058. Similarly, The Student *t*-test accepts the null hypothesis of equality of the means when applied to first cumulant data from healthy and unhealthy inspirations as the associated *p*-value is 0.8869. Figure 8 exhibits the second cumulant boxplot. The Student *t*-test leads to the rejection of the null hypothesis of equality of the means when applied to the second cumulant data from healthy and unhealthy expirations as the associated *p*-value is 1.4661 × 10^−7^. Similarly, The Student *t*-test leads to the rejection of the null hypothesis of equality of the means when applied to second cumulant data from healthy and unhealthy inspirations as the associated *p*-value is 1.0421 × 10^−6^. Figure 9 shows the third cumulant boxplot. The Student *t*-test rejects the null hypothesis of equality of the means when applied to the third cumulant data from healthy and unhealthy expirations as the associated *p*-value is 0.0053. Similarly, the Student *t*-test leads to the rejection of the null hypothesis of equality of the means when applied to the third cumulant data from healthy and unhealthy inspirations as the associated *p*-value is 2.1317 × 10^−6^. In short, the results from statistical tests (Figure 6) suggest that multifractal characteristics of expiration and inspiration signals are different across healthy and unhealthy conditions. In addition, linear and nonlinear components of cepstra are statistically different across healthy and unhealthy infant cry signals (Figure 7, Figure 8 and Figure 9).

In summary, we examined the multifractal structure in the cepstra of cries in healthy and unhealthy infants during expiration and inspiration. The results can be presented as follows:

1. All infant cry records exhibit evidence of multifractal properties according to estimated multifractal spectra *D*(*h*) and scaling exponent functions ζ(*q*).
2. The mean of spectrum *D*(*h*) is larger under healthy conditions than under unhealthy conditions. In addition, expiration and inspiration signals exhibit more complexity under healthy conditions than under unhealthy conditions.
3. Multifractal characteristics as represented by the first, second, and third cumulants in expiration, and inspiration signals are statistically different across healthy and unhealthy conditions.

Our statistical analysis of baby cry records by means of wavelet leaders revealed interesting findings that might be promising in the medical milieu. Indeed, the findings are statistically significant. The bottom line is that both healthy and unhealthy infant cry records are characterized by multifractal structures. More importantly, the cepstra of healthy records exhibit more fractality and complexity than the cepstra of unhealthy records. Recall that some recent studies have shown that healthy biomedical signals are characterized by larger complexity compared to unhealthy ones. For instance, the multifractal spectrum of electrocardiograms was found to be small during periods of atrial fibrillation than during various rhythms, including normal ones [11]. Likewise, normal electrocardiograms exhibit larger complexity than abnormal ones [39]. In this regard, the reduced complexity in abnormal biomedical signals can be explained by the presence of malfunctions in the organs and systems of the body [39]. Such differences can help to understand the complex system of human physiology, specifically in terms of infant cries in healthy and unhealthy conditions. In this regard, multifractal analyses using wavelet leaders may be integrated within a computer to improve the correctness of medical diagnosis for better appropriate treatment.

## 4. Conclusions

It is well known that multifractal properties exist in various biomedical signals, including EEG, ECG, magnetic resonance images and brainstem volume, mammograms, bone radiographic images, retina digital images, dental implant ultrasonic signal, and liver tissue images. However, no work has been devoted to examining the presence of multifractals in baby cry records. Indeed, this investigation could help to understand the physiological aspects of such biomedical signals across healthy and unhealthy babies.

We studied the multifractal behavior in the cepstrum representation of healthy and unhealthy infant cry signals by applying the technique of wavelet leaders. The empirical results demonstrate that both expiration and inspiration signals show strong evidence of multifractal properties under healthy and unhealthy conditions. In addition, for both expiration and inspiration sets, healthy signals exhibit a higher degree of multifractality than unhealthy ones. Our findings could help to understand the multifractal nature in cepstra of healthy and pathological infant cry signals for the better characterization of pathologies. We have not explored the sources of multifractality in the cepstrum domain. However, such investigation is left for future work and will be applied to original infant cry signals. In addition, our future work will also consider the combination of multifractal characteristics and machine learning for the classification of baby cry records.

## References

[1] Müller A., Kraemer J.F., Penzel T., Bonnemeier H., Kurths J., Wessel N. (2016). Causality in physiological signals. Physiol. Meas..

[2] Peitgen H.-O., Jürgen H., Saupe D. (1992). Chaos and Fractals: New Frontiers of Science.

[3] Souza França L.G., Vivas Miranda J.G., Leite M., Sharma N.K., Walker M.C., Lemieux L., Wang Y. (2018). Fractal and Multifractal Properties of Electrographic Recordings of Human Brain Activity: Toward Its Use as a Signal Feature for Machine Learning in Clinical Applications. Front. Physiol..

[4] Zorick T., Mandelkern M.A. (2013). Multifractal detrended fluctuation analysis of human EEG: Preliminary investigation and comparison with the wavelet transform modulus maxima technique. PLoS ONE.

[5] Zhang Y., Zhou W., Yuan S. (2015). Multifractal Analysis and Relevance Vector Machine-Based Automatic Seizure Detection in Intracranial EEG. Int. J. Neural Syst..

[6] Lahmiri S. (2018). An accurate system to distinguish between normal and abnormal electroencephalogram records with epileptic seizure free intervals. Biomed. Signal Processing Control.

[7] Lahmiri S., Shmuel A. (2018). Accurate classification of seizure and seizure-free intervals of intracranial EEG signals from epileptic patients. IEEE Trans. Instrum. Meas..

[8] Lahmiri S. (2018). Generalized Hurst exponent estimates differentiate EEG signals of healthy and epileptic patients. Phys. A.

[9] Gaurav G., Anand R.S., Kumar V. (2021). EEG based cognitive task classification using multifractal detrended fluctuation analysis. Cogn. Neurodyn..

[10] Zebende G.F., Oliveira Filho F.M., Leyva Cruz J.A. (2017). Auto-correlation in the motor/imaginary human EEG signals: A vision about the FDFA fluctuations. PLoS ONE.

[11] Gadhoumi K., Do D., Badilini F., Pelter M.M., Hu X. (2018). Wavelet leader multifractal analysis of heart rate variability in atrial fibrillation. J. Electrocardiol..

[12] Orozco-Duque A., Novak D., Kremen V., Bustamante J. (2015). Multifractal analysis for grading complex fractionated electrograms in atrial fibrillation. Physiol. Meas..

[13] Aguilar-Molina A.A., Angulo-Brown F., Muñoz-Diosdado A. (2019). Multifractal Spectrum Curvature of RR Tachograms of Healthy People and Patients with Congestive Heart Failure, a New Tool to Assess Health Conditions. Entropy.

[14] Reyes-Manzano C.F., Lerma C., Echeverría J.C., Martínez-Lavin M., Martínez-Martínez L.A., Infante O., Guzmán-Vargas L. (2018). Multifractal Analysis Reveals Decreased Non-linearity and Stronger Anticorrelations in Heart Period Fluctuations of Fibromyalgia Patients. Front. Physiol..

[15] Lin D.C., Sharif A. (2010). Common multifractality in the heart rate variability and brain activity of healthy humans. Chaos.

[16] Lahmiri S. (2017). Glioma detection based on multi-fractal features of segmented brain MRI by particle swarm optimization techniques. Biomed. Signal Processing Control.

[17] Lahmiri S., Boukadoum M. (2014). New approach for automatic classification of Alzheimer’s disease, mild cognitive impairment and healthy brain magnetic resonance images. Healthc. Technol. Lett..

[18] Rohini P., Sundar S., Ramakrishnan S. (2020). Differentiation of early mild cognitive impairment in brainstem MR images using multifractal detrended moving average singularity spectral features. Biomed. Signal Processing Control.

[19] Gerasimova-Chechkina E., Toner B., Marin Z., Audit B., Roux S.G., Argoul F., Khalil A., Gileva O., Naimark O., Arneodo A. (2016). Comparative Multifractal Analysis of Dynamic Infrared Thermograms and X-Ray Mammograms Enlightens Changes in the Environment of Malignant Tumors. Front. Physiol..

[20] Gerasimova-Chechkina E., Toner B., Marin Z., Audit B., Roux S.G., Argoul F., Khalil A., Gileva O., Naimark O., Arneodo A. (2014). Wavelet-based multifractal analysis of dynamic infrared thermograms to assist in early breast cancer diagnosis. Front. Physiol..

[21] Borowska M., Bębas E., Szarmach J., Oczeretko E. (2019). Multifractal characterization of healing process after bone loss. Biomed. Signal Processing Control.

[22] Wang J., Shao W., Kim J. (2020). Combining MF-DFA and LSSVM for retina images classification. Biomed. Signal Processing Control.

[23] Scala I., Rosi G., Nguyen V.-H., Vayron R., Haiat G., Seuret S., Jaffard S., Naili S. (2018). Ultrasonic characterization and multiscale analysis for the evaluation of dental implant stability: A sensitivity study. Biomed. Signal Processing Control.

[24] Oprić D., Stankovich A.D., Nenadović A., Kovačević S., Obradović D.D., de Luka S., Nešović-Ostojić J., Milašin J., Ilić A.Ž., Trbovich A.M. (2020). Fractal analysis tools for early assessment of liver inflammation induced by chronic consumption of linseed, palm and sunflower oils. Biomed. Signal Processing Control.

[25] Wendt H., Abry P. (2007). Multifractality Tests Using Bootstrapped Wavelet Leaders. IEEE Trans. Signal Processing.

[26] Peng C.K., Buldyrev S.V., Havlin S., Simons M., Stanley H.E., Goldberger A.L. (1994). Mosaic organization of DNA nucleotides. Phys. Rev. E.

[27] Di Matteo T. (2007). Multi-scaling in finance. Quant. Financ..

[28] Matikolaie F.S., Tadj C. (2020). On the use of long-term features in a newborn cry diagnostic system. Biomed. Signal Processing Control.

[29] Alaie H.F., Abou-Abbas L., Tadj C. (2016). Cry-based infant pathology classification using GMMs. Speech Commun..

[30] Hariharan M., Sindhu R., Vijean V., Yazid H., Nadarajaw T., Yaacob S., Polat K. (2018). Improved binary dragonfly optimization algorithm and wavelet packet based non-linear features for infant cry classification. Comput. Methods Programs Biomed..

[31] Orlandi S., Garcia C.A.R., Bandini A., Donzelli G., Manfredi C. (2016). Application of Pattern Recognition Techniques to the Classification of Full-Term and Preterm Infant Cry. J. Voice.

[32] Kheddache Y., Tadj C. (2019). Identification of diseases in newborns using advanced acoustic features of cry signals. Biomed. Signal Processing Control.

[33] Kheddache Y., Tadj C. (2015). Resonance frequencies behavior in pathologic cries of newborns. J. Voice.

[34] Childers D.G., Skinner D.P., Kemerait R.C. (1977). The Cepstrum: A Guide to Processing. Proc. IEEE.

[35] Oppenheim A.W. (2010). Discrete-Time Signal Processing.

[36] Travieso C.M., Alonso J.B., del Pozo M., Ticay J.R., Castellanos-Dominguez G. (2014). Building a Cepstrum-HMM kernel for Apnea identification. Neurocomputing.

[37] Lahmiri S., Tadj C., Gargour C. (2021). Biomedical diagnosis of infant cry signal based on analysis of cepstrum by deep feedforward artificial neural networks. IEEE Instrum. Meas. Mag..

[38] Lahmiri S., Tadj C., Gargour C., Bekiros S. (2022). Deep learning systems for automatic diagnosis of infant cry signals. Chaos Solitons Fractals.

[39] Shekatkar S.M., Kotriwar Y., Harikrishnan K.P., Ambika G. (2017). Detecting abnormality in heart dynamics from multifractal analysis of ECG signals. Sci. Rep..

